# Using selective lung injury to improve murine models of spatially heterogeneous lung diseases

**DOI:** 10.1371/journal.pone.0202456

**Published:** 2019-04-03

**Authors:** Andrew J. Paris, Lei Guo, Ning Dai, Jeremy B. Katzen, Priyal N. Patel, G. Scott Worthen, Jacob S. Brenner

**Affiliations:** 1 Department of Medicine, Division of Pulmonary, Allergy and Critical Care Medicine, Perelman School of Medicine, University of Pennsylvania, Philadelphia, Pennsylvania, United States of America; 2 Kunming National High-level Biosafety Research Center, Institute of Medical Biology, Chinese Academy of Medical Science, Kunming, Yunnan, China; 3 Department of Pediatrics, Division of Neonatology, Perelman School of Medicine, University of Pennsylvania, Philadelphia, Pennsylvania, United States of America; 4 Department of Systems Pharmacology and Translational Therapeutics and Center for Translational Targeted Therapeutics and Nanomedicine, Perelman School of Medicine, University of Pennsylvania, Philadelphia, Pennsylvania, United States of America; 5 Penn Center for Pulmonary Biology, Perelman School of Medicine, University of Pennsylvania, Philadelphia, Pennsylvania, United States of America; Ann and Robert H Lurie Children's Hospital of Chicago, Northwestern University, UNITED STATES

## Abstract

Many lung diseases, such as the acute respiratory distress syndrome (ARDS), display significant regional heterogeneity with patches of severely injured tissue adjacent to apparently healthy tissue. Current mouse models that aim to mimic ARDS generally produce diffuse injuries that cannot reproducibly generate ARDS’s regional heterogeneity. This deficiency prevents the evaluation of how well therapeutic agents reach the most injured regions and precludes many regenerative medicine studies since it is not possible to know which apparently healing regions suffered severe injury initially. Finally, these diffuse injury models must be relatively mild to allow for survival, as their diffuse nature does not allow for residual healthy lung to keep an animal alive long enough for many drug and regenerative medicine studies. To solve all of these deficiencies in current animal models, we have created a simple and reproducible technique to selectively induce lung injury in specific areas of the lung. Our technique, catheter-in-catheter selective lung injury (CICSLI), involves guiding an inner catheter to a particular area of the lung and delivering an injurious agent mixed with nanoparticles (fluorescently and/or radioactively labeled) that can be used days later to track the location and extent of where the initial injury occurred. Furthermore, we demonstrate that CICSLI can produce a more severe injury than diffuse models, yet has much higher survival since CICSLI intentionally leaves lung regions undamaged. Collectively, these attributes of CICSLI will allow investigators to better study how drugs act within heterogeneous lung pathologies and how regeneration occurs in severely damaged lung tissue, thereby aiding the development of new therapies for ARDS and other heterogenous lung diseases.

## Introduction

Acute respiratory distress syndrome (ARDS) is characterized by bilateral non-cardiogenic pulmonary edema that appears as heterogeneously distributed patchy pulmonary infiltrates on chest x-rays and CT scans[1–3]. While this heterogeneous distribution has been noted since the first descriptions of ARDS[4], it has been very difficult to study the consequences of regional heterogeneity of ARDS in experimental animal models. Animal models intended to model ARDS, called acute lung injury (ALI) models, such as intra-tracheal or instilled toxins or intravenous toxins produce a *diffuse* lung injury[5, 6]. When these models do produce heterogeneity[7], such as in the intra-nasal influenza model, the patchiness has very high inter-individual variability, largely limiting systematic study of the effects of patchy injury[8]. Furthermore, because of the diffuse nature of such experimental ALI models, the injuries must be restrained to allow the animals to survive long enough for many analyses, such as for lung regeneration studies[7, 9].

These diffuse ALI models are particularly problematic for the two important fields of translational medicine aiming to ameliorate ARDS: development of therapeutics and regenerative medicine[10–15]. With regards to pharmaceutical development, investigators need to demonstrate that potential therapeutics for ARDS reach the patches of inflamed tissue (not just healthy lung regions), but diffuse ALI models do not allow such analyses because of their diffuse nature [15]. Thus, with current diffuse ALI models we cannot determine the intra-pulmonary distribution of small molecule drugs, nano-scale drug delivery vehicles, inhaled drugs, or cell therapies. In the field of regenerative medicine, investigators looking at healthy tissue at the end of an experiment need to know if an apparently healed lung region was actually damaged, or if its healed appearance is due to having never been injured. Since regenerative medicine requires analysis days to weeks after the injury, it is essential to be able to know the precise location and severity of the initial lung lesions, even at long time points after injury[7, 9, 16]. Thus, for the development of ARDS therapeutics and post-ARDS lung regeneration treatments, the current diffuse ALI models leave room for optimization.

To solve these problems, we have created a very simple and reproducible system for creating and precisely tracking a heterogeneous and severe acute lung injury (ALI). In this system, termed catheter-in-catheter selective lung injury (CICSLI), mice are endotracheally intubated with a catheter[17], followed by insertion of a smaller catheter directed either into a single lung or single lobe, which is determined by the length of the smaller catheter. Subsequently, a solution is instilled into the smaller catheter that contains an injurious insult that has a low concentration of polymeric nanoparticles (labeled with fluorescence and/or radioactive moieties). This produces a relatively higher degree of lung injury than would be possible in a diffuse injury caused by the same agent while simultaneously ensuring excellent animal survival. Further, the nanoparticles allow tracing, several days later, of precisely where the injurious insult occurred, allowing fine determination of which regions received the initial injury, and correlating that with therapy distribution and lung regeneration assays.

## Materials and methods

### Mice

All mice were housed in SPF conditions in an animal facility at the Children’s Hospital of Philadelphia. WT C57BL/6J mice (strain 000664 from the Jackson Laboratory, Bar Harbor, ME) aged 8–12 weeks were used for experiments (18-25g). Both male and female mice were used in equal proportions. All mouse protocols were approved by the IACUC at the Children’s Hospital of Philadelphia. Unless otherwise specified, we used 4 mice per time point per treatment condition in each experiment. Mice were housed and used in accordance with institutional and AALAC guidelines.

### Injury model

Sedated mice were intubated using a 20G angiocatheter (BD catalog #381434) using a previously described technique[17]. The mice were then placed in the right or left lateral recumbent position with the mouse’s head tilted upward and a polyethylene 10 (PE-10) catheter (BD catalog #427400) was directed into the right main stem bronchus. For the acid injury model, we instilled 2 μL/g of osmotically balanced 0.1N HCl into the right lung through the PE-10 catheter.

### Instillation of LPS

Lipopolysaccharide B4 (Sigma catalog # L2630) was selectively instilled into mouse airways, as described above. Each mouse was instilled with 1 mg/kg of LPS. For the LPS data in this paper, we used a pre-specified, objective, quality-control metric to ensure that all mice had received a true localized injury. To accomplish this, before analyzing each mouse, we weighed all the lobes of the lungs. We then determined the degree of localization of LPS injury by comparing the ratio of the weight of the superior lobe (the lobe intended for LPS instillation) divided by the weight of the left lung (the lobe that should get the least LPS). For naïve mice, this S:L (superior lobe: left lung) weight ratio was 0.55 +/- 0.02 (n = 6). By comparison, in a cohort of n = 13 mice in which LPS was instilled into the superior lobe by the CICSLI technique and all the mice had, on visual inspection, injury confined to the superior lobe, the S:L weight ratio was 1.13 +/- 0.04, with a range of 0.94 to 1.28. Therefore, we developed as a standard for further experiments a cut-off S:L weight ratio of 0.9. Thus, any mice that received a CICSLI injury and had an S:L weight ratio of < 0.9 were removed before any further analyses. This pre-specified, objective, quality-control metric aims to ensure that all mice had received a true localized injury. Among experienced practitioners of the CICSLI technique, those who had previously performed the technique a minimum of 20 times, less than 20% of mice were removed by that objective, pre-specified criteria.

### Nanoparticle-based tracking of the location of instillate into airways

Polymeric nanoparticles (NPs) were purchased from Bangs Laboratories, Inc. For tracking where the NPs localized by immunofluorescence days after acid instillation, we used NPs that were 1000 nanometer (nm) in diameter and composed of polystyrene with the fluorophore FlashRed (similar spectrum to Cy5) covalently attached (Bangs Labs Catalog #FSFR004). For radiotracing where the instillate localized, we used 200-nm polystyrene conjugated to FlashRed and with surface carboxylate groups (Catalog #FSFR002). The surface carboxylation allowed for conjugation to radiolabeled proteins, as described below.

### Radiolabeling nanoparticles

Rat IgG (ThermoFisher catalog #31933) was labeled with I-125 via Pierce Iodination Beads (ThermoFisher catalog # 28665). We then conjugated the IgG to nanoparticles using our published protocol[15]. In brief, 100 μL of polystyrene NPs were buffer exchanged with Zeba Spin Desalting Columns, 7K MWCO, 0.5 mL (ThermoFisher catalog # 89882), exchanging for 50 mM MES buffer at pH 5.2, finally putting the buffer-exchanged beads into 1.5 mL Eppendorf tubes. Next N-Hydroxysulfosuccinimide (“sulfo-NHS”; Sigma catalog #56485) was added to a final concentration of 0.275 mg/ml and incubated for 3 minutes at room temperature (RT). Next N-(3-Dimethylaminopropyl)-N′-ethylcarbodiimide hydrochloride (“EDAC”; Sigma catalog # E7750) was added to a final concentration of 0.1 mg/ml and incubated for 10–15 minutes at RT. Next 114 ug of I-125-labeled rat IgG was added (giving 200 antibody molecules per bead) and incubated for 2 to 4 hours at RT on a vortex/shaker at low speed. 1 mL MES buffer was added to dilute free antibody, followed by centrifuge at 12,000g x 3 min to pellet the IgG-conjugated NPs. The IgG-NP pellet was resuspended in 200 uL of PBS + 0.05% bovine serum albumin (BSA) buffer. Immediately before use, the NPs were sonicated with a probe / tip sonicator (Qsonica Q55 Sonicator, Cole-Parmer #UX-04712-51) for three 3-second pulses at 30% maximum power.

### Radiolabeled albumin tracing

BSA was I-125-labeled as described for IgG above (Perkin-Elmer catalog # NEZ033005MC). Mice were given selective instillation of LPS, followed 20 hours later by intravenous injection of 1 x 10^6^ counts per minute (cpm) of I-125-BSA, followed by sacrifice 4 hours later. The right ventricle was then flushed with 10 mL of PBS to flush out the pulmonary vasculature of residual blood. The lobes of the lungs were then individually removed from chest cavity and measured for I-125 levels in a gamma counter (Perkin Elmer Wizard-2, catalog # 2470–0020).

### Evans blue determination of capillary leak

Mice were given lobar LPS. Twenty-four hours later, mice were injected IV with Evans Blue at 30 mg/kg, followed 2 hours later by perfusing the right ventricle with 10 mL of PBS + 10mM EDTA. The lung lobes were individually dissected from the chest and a photograph was taken of them.

### CT scanning of mice

Mice were given lobar LPS. Twenty-four hours later, under general anesthesia a tracheostomy was created and a 20-gauge peripheral IV catheter was placed into the tracheostomy. The mouse was then immediately sacrificed via overdose of ketamine. Before the development of post-mortem atelectasis, the tracheostomy tube was insufflated with 300 uL of air, then the tube was removed while a ligature was cinched around the trachea, thus ensuring the lungs remained filled with air. Immediately after sacrifice, the mouse’s body was put into a small animal microCT scanner made by (ImTek MicroCAT II MicroCT Scanner), housed at the University of Pennsylvania’s Small Animal Imaging Facility.

## Results

### CICSLI is a simple and traceable method for selectively injuring murine lungs

**Fig 1:**
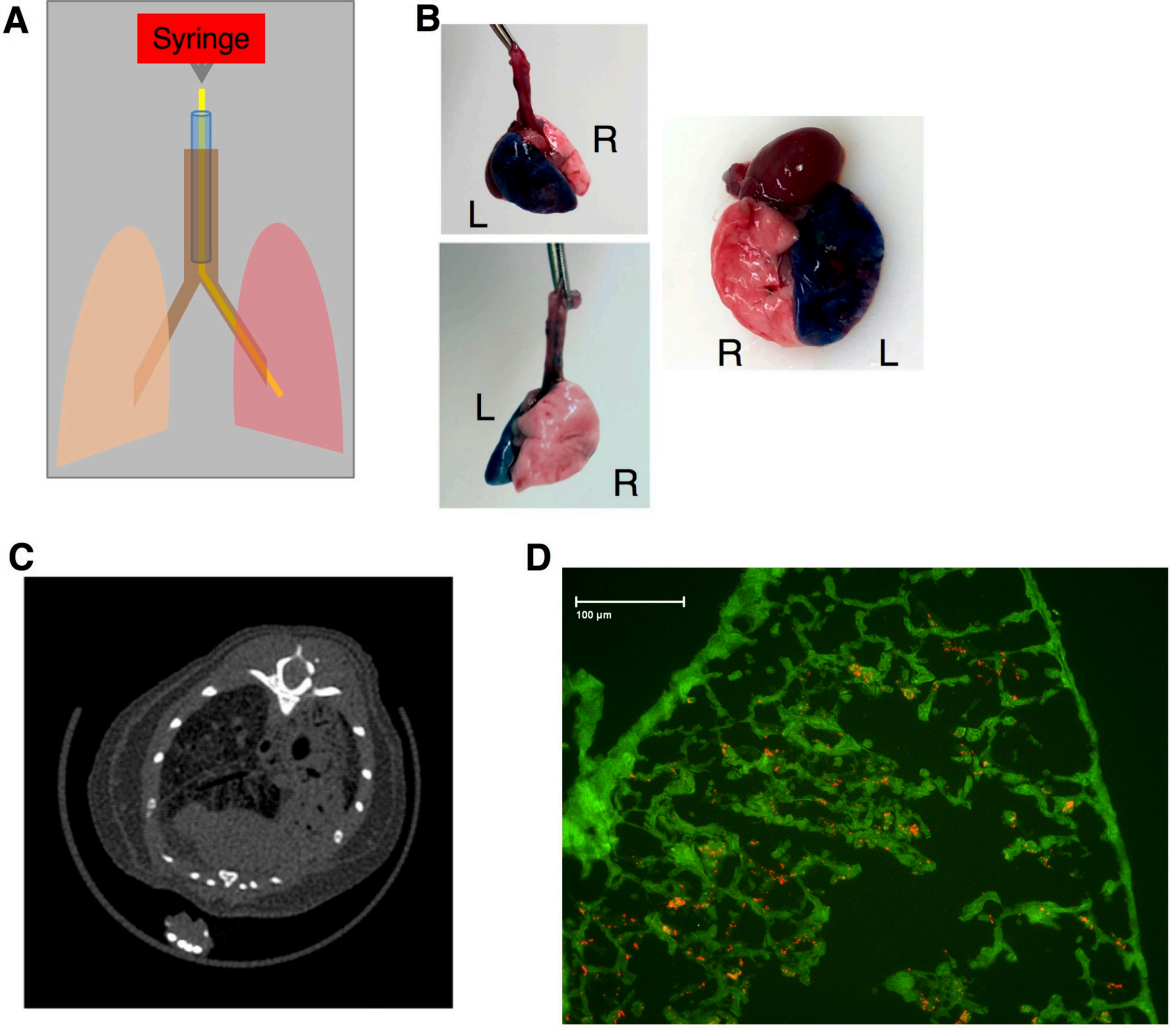
Unilateral lung injury created by selectively instilling acid. A) Procedure for single lung acute injury. The mouse was sedated, followed by orotracheal intubation with a 20G angiocatheter (blue) that terminates in the trachea (brown). The mouse is then placed in the left lateral recumbent position and a 51mm PE10 catheter (yellow) is advanced into the inferior lung (the left lung in this example). A syringe is attached to the catheter using a 0.5 inch 30G needle and a specified amount of liquid is selectively delivered to the inferior lung (red). B) Healthy mice underwent selective instillation of methylene blue as detailed in A. The mouse was euthanized 5 minutes after instillation. Selective injury of the left lung was verified visually. C) Selective instillation of 0.1N osmotically balanced hydrochloric acid into the right lung was performed 24 hours before euthanizing the mice and subjecting them to microCT scans. CT imaging shows parenchymal injury exclusively on the right side. D) Mice underwent single lung instillation of acid containing 1 μM FlashRed-labeled polystyrene beads, followed by euthanasia 72 hours later. Frozen sections of embedded injured lung tissue demonstrated persistence of beads at 72 hours post-injury. Green represents tissue autofluorescence.

We developed a straightforward and minimally invasive technique for instilling liquid into a mouse’s lung. The procedure starts with intubating the mouse using a previously established method of orotracheal intubation using a 20g angiocatheter[17]. Once the angiocatheter has been advanced into the mouse’s trachea, we then insert polyethylene 10 tubing (54 mm) attached to a 1mL syringe via a 30G needle (Fig 1A). For the purpose of demonstrating the method in the left lung we used a 2uL per gram of methylene blue followed by 200ul of air. The mouse was placed on in the left lateral recumbent position before slowly instilling the methylene blue. Afterwards, the mouse was placed in the supine position and the lungs were extracted and photographed to demonstrate the that this method can achieve selective instillation of a liquid into a living mouse (Fig 1B).

To demonstrate that this method can be used to induce selective lung injury we repeated the procedure outlined in Fig 1A but instead placed the mouse in the right lateral recumbent position in order to guide the PE 10 catheter into the right lung and instilled 2.5ul per gram of 0.1N hydrochloric acid into the mouse’s right lung. A CT scan performed one day later showed selective injury of the right lung, indicating that we can safely induce a radiographically apparent injury without causing death (Fig 1C). Because this method is ideal for studying lung repair we wanted to find a way to distinguish where the injury occurred. Ordinarily, it may not be possible to distinguish between uninjured and properly regenerated lung tissue, therefore we sought to mark where injury had occurred by including 1uM of FlashRed-labeled polystyrene beads. Frozen section of the injured right lung demonstrate that the beads are readily apparent even when delivered in a solution containing 0.1N hydrochloric acid (Fig 1D). No beads were visible in the contralateral, uninjured, lung.

### CICSLI increases local tissue injury and decreases mortality, relative to diffuse instillation

**Fig 2:**
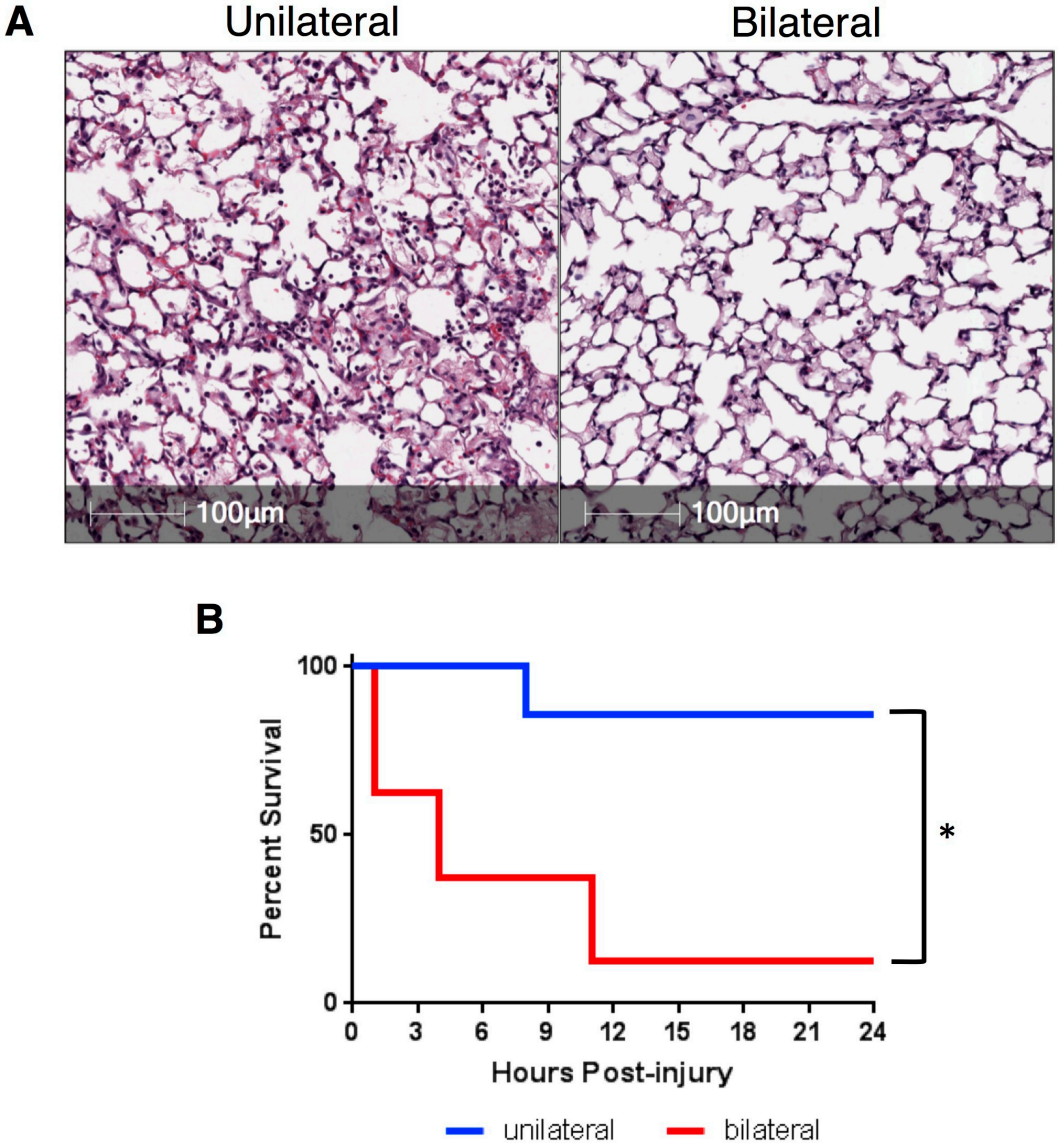
Unilateral lung injury increases injury while improving survival. A) H&E staining of lung sections taken from C57BL/6 mice 24 hours after undergoing unilateral (injured area shown) or bilateral lung injury demonstrates significantly increased accumulation of cellular infiltration and protein-rich edema fluid when compared to the lung that underwent bilateral (intratracheal) lung injury. B) We subjected mice to right unilateral (n = 7) and bilateral (n = 8) lung injury. Mice were evaluated on an hourly basis and were euthanized if a blinded observer determined that the mouse was obtunded. Differences in mortality were significant, *p* = 0.0055, when compared using a log-rank (Mantel-Cox) test.

To ascertain the relative difference in tissue injury between unilateral and diffuse lung injury, we instilled 2.5ul/g of 0.1N hydrochloric acid into the lungs of C57BL/6 mice using our selective method (Fig 1) or intratracheally, meaning without using the PE10 catheter. We observed a relatively more severe lung injury in the selective group with a markedly increased cellular infiltrate and hyaline membrane formation. The mice that received bilateral lung injury had less cellular infiltrate (Fig 2A). Although intratracheal administration produced a more bland-appearing injury, the survival at twenty-four hours was significantly lower in the mice that had bilateral lung injury relative to those that had unilateral injury (Fig 2B). These data reveal a paradox in which selectively injuring lungs increases histological injury while decreasing mortality.

### Selectively injuring a single lobe is possible with injury remaining isolated

**Fig 3:**
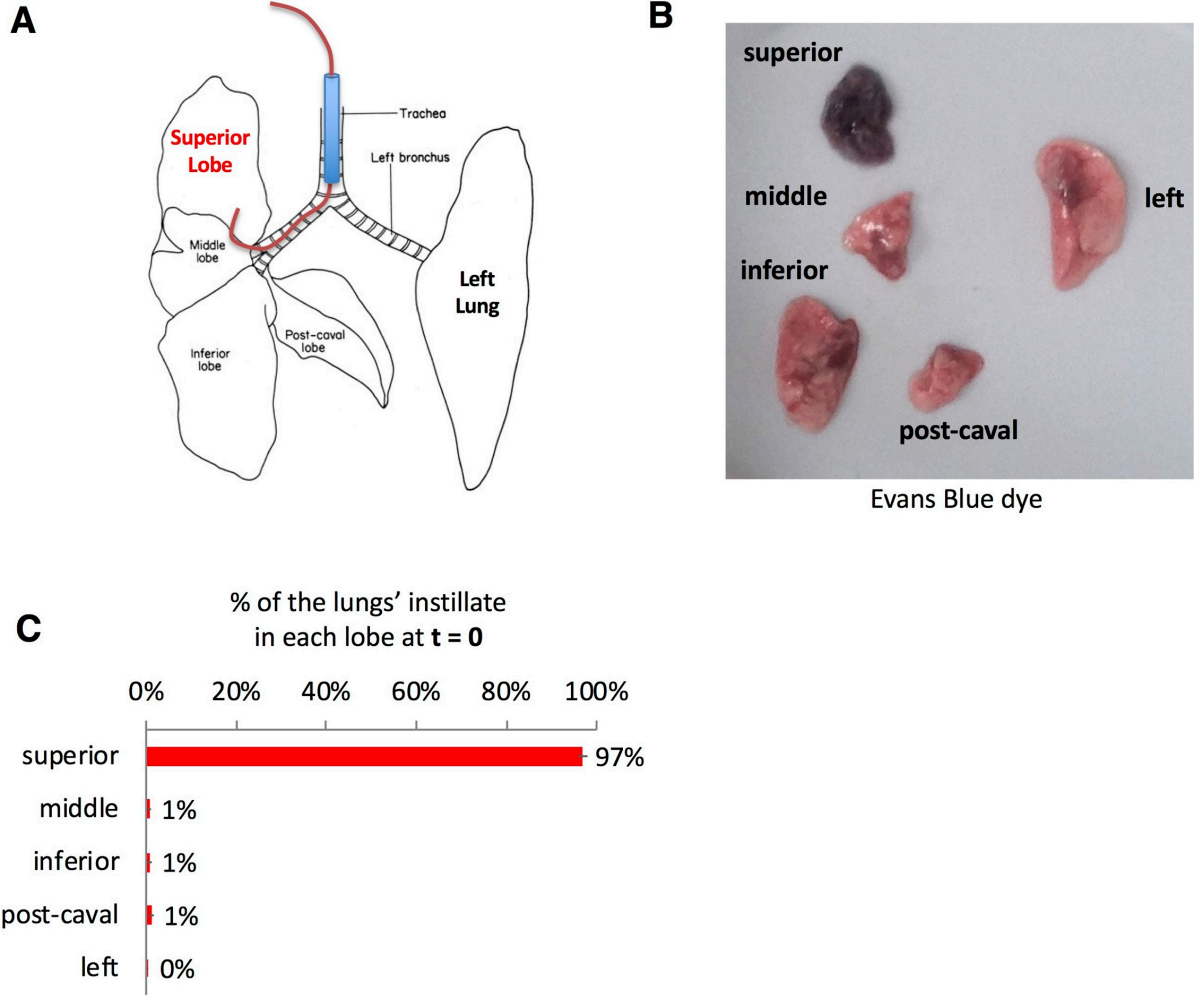
Single-lobe injury created by a longer instillation catheter. A) Mice were orotracheally intubated with a 20G angiocatheter terminating in the trachea (brown). The mice were then placed in the left lateral recumbent position and a 59mm PE10 catheter was inserted into the endotracheal catheter (note this is 8mm longer than the catheter used for acid aspiration experiments). The mice were then kept in left lateral recumbent for 20 minutes after instillation. B) Mice were instilled as in A, with the instillate containing 1mg/kg of LPS, 24 hours later, Evans Blue dye was injected followed by sacrifice 2 hours later, with visible photography of the lobes. C) Mice were treated as in B, but into the LPS mixture we added 100-nanometer diameter polystyrene beads that were covalently coated with I-125-labeled-rat-IgG. Immediately after instillation, the mice were sacrificed, followed by measurement of each lobe in a gamma counter. Each data point represents mean ± s.e.m (n = 3). * p<0.0001, one-way ANOVA.

Data shown in Figs 1 and 2 demonstrate that instilling agents through a catheter that is longer than the endotracheal tube can help selectively injure the left (Fig 1A and 1B) or right (Fig 1C) lung. Based on these data we hypothesized that using a longer catheter and smaller volume of fluid could selectively injure a single lobe of the right lung. To test this hypothesis, we repeated the experimental design shown in Fig 1 but used a slightly longer catheter– 59mm PE 10 catheter, instilling 50 uL total of liquid (Fig 3A). Using Evan's blue dye, we show that the longer catheter is able to selectively instill fluid into the right upper lobe (RUL) (Fig 3B). The immediate specificity of selectively instilling lipopolysaccharide (LPS) into the right upper lobe was assayed by co-instilling I-125-labelled-IgG that was bound to 100nm polystyrene beads along with the LPS. We observed that 97% of the radiolabeled albumin was detected in the RUL, quantifying the selectiveness of this approach (Fig 3C). To determine if the initial instillation remained isolated in the right upper lobe we repeated the same test with I-125-labeled IgG co-administered with LPS and found that 92% of the isotope was detected in the right upper lobe 24 hours after selectively instilling LPS in the right upper lobe, suggesting that the selective lung injury did not spread to other lobes.

### Selective lung injury changes regional lung physiology

**Fig 4:**
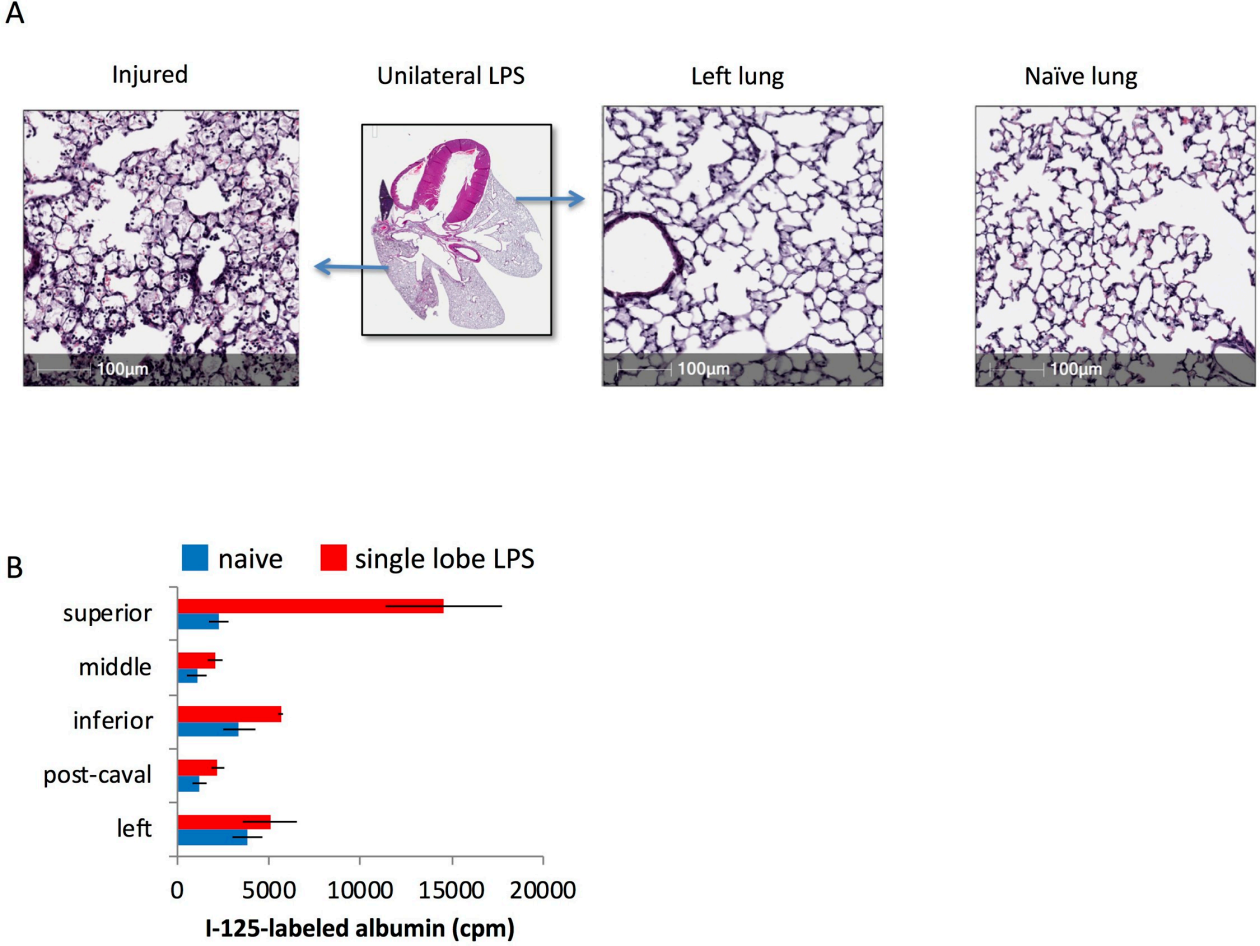
In single-lobe lung injury, some pathological phenotypes are restricted to injured lobes. A) Mice underwent single-lobe LPS instillation as in Fig 3, followed by sacrifice 24 hours later, and then H&E staining of the lungs. Comparison is shown to a naïve mouse. B) Mice underwent single-lobe LPS instillation, followed by injection of I-125-labeled albumin 24 hours later, followed by sacrifice, perfusion of the pulmonary arteries with 5 mL of PBS, and then gamma counting of the lobes. Note that the lobe into which LPS was instilled (the superior) has a greater capillary permeability than the other lobes. Each data point represents the mean ± s.e.m (n = 3). * p<0.0001, one-way ANOVA, followed by pairwise comparison for each lobe, comparing naïve vs single lobe LPS, with all lobes having non-significant differences except the superior lobe.

Hematoxylin and eosin (H&E) staining of a lung following lung injury revealed a spectrum of tissue damage within the injured lung, which we attribute to uneven distribution of the LPS within the injured lobe. Importantly, we did not observe any areas of uninjured tissue within the injured lobe (Fig 4A). Although selective injury was able to induce lobe-specific tissue damage (Figs 1C and 4A) we were uncertain if a seminal physiologic response to injury was also regional or if the severe injury would induce systemic changes in all lobes. To assay for vascular leak, we injected I-125-labeled albumin via retro-orbital injection 24 hours after inducing selective lung injury. When compared to uninjured mice we found that only the right upper lobe had a significantly increased amount of radiolabeled albumin (Fig 4B). These data suggest that selective injury can recapitulate lobe-specific tissue damage and physiologic change.

## Discussion

Developing and testing new therapeutics for lung diseases starts with using faithful pre-clinical models that recapitulate key aspects of human lung disease[15, 18]. Intratracheal administration of injurious agents or inducing non-specific systemic inflammation either create bland injury that falls short of the severe injury that often produces the greatest mortality or it creates such a severe injury that studying long-term regeneration is not possible. Furthermore, inducing regional physiologic changes can help us understand how drugs will be distributed functionally in a patient with a mix of injured and healthy tissues[15], which is the case in most human lung diseases[3, 19–21].

In this paper we describe a simple and reproducible technique to selectively injure specific parts of the lung. By combining a catheter-within-a-catheter technique and positioning a mouse in the right or left lateral recumbent position we can easily direct a lung injury to the right or left lung. Further lengthening the inner catheter facilitates selective injury of the right upper lobe. Inducing injury in this way is the major innovation of our model because of its ability to produce a histologically severe yet simultaneously more survivable injury. This has allowed us to study regeneration following a tissue injury that would otherwise be unsurvivable[22].

We considered that using these models to study lung regeneration would be challenging as newly regenerated lung tissue would appear histologically identical to the uninjured lung. For the CICSLI models, we chose as our instillates LPS and acid, since the former models the two most common stimuli for ARDS (sepsis and pneumonia), and the latter models the third most common cause of ARDS (aspiration). Although lineage tracing[23] of alveolar epithelial progenitors could be used to overcome this challenge, there is no way to label all progenitor populations[7, 9, 16, 24, 25], meaning that regenerated lung tissue could still resemble uninjured lung even when using lineage tracing. Labeling the injured area is thus an essential second innovation that we are reporting. We show here that co-administration of innocuous fluorescently-labeled beads or radiolabeled albumin will persist as validated markers of where injury occurred. Although we show that fluorescently-labeled beads will persist for at least 72 hours, more studies are needed to know if they will persist for longer periods of time.

We have also shown that our selective lung injury model is useful for inducing selective changes in lung permeability. This third innovation recapitulates key heterogeneity that occurs clinically [2, 3, 26] and has important implications for evaluating candidate drugs. For example, we have already demonstrated that CICSLI induces regional changes in drug delivery that have helped us understand how to selectively deliver drugs to injured lungs while avoiding normal tissue[15].

Although other groups have published reports utilizing selective lung injury[27, 28] we believe that this is the first systematic approach to delineating the methodology and interrogating the physiology though the lens of developing better pre-clinical mouse models of lung disease. Further work is needed to determine best-practices for histologically scoring heterogenous lung injuries and assaying lung function. Furthermore, technological advances, such as the recently described mouse bronchoscope[29], may facilitate repeat instillations and sequential sampling. Nevertheless, this report details a simple, low-cost, and low-labor method that can be easily employed that can dramatically improve the study of ARDS.

Notably, the CICSLI technique can be used to prepare not just ARDS models, but perhaps other heterogeneous lung diseases. Numerous other lung diseases are modeled by intra-tracheal instillations: pneumonia with bacteria[30], idiopathic pulmonary fibrosis with bleomycin[31], and asthma with airway irritants[32]. Each of these human diseases is very spatially heterogeneous and, as such, would benefit from being modeled using CICSLI. Additionally, CICSLI could be done with new agents, such as transfection with gene therapy and CRISPR reagents, to create new pathology models.

In summary, CICSLI provides a simple, easy, quick and inexpensive method to improve key features of pre-clinical lung disease models, which should aid the development of new drugs and regenerative medicine therapies for many lung diseases.
